# Tissue remodeling after ocular surface reconstruction with denuded amniotic membrane

**DOI:** 10.1038/s41598-018-24694-4

**Published:** 2018-04-23

**Authors:** Jing Jie, Jie Yang, Hui He, Jianlan Zheng, Wenyan Wang, Liying Zhang, Zhiyuan Li, Jingyao Chen, M. Vimalin Jeyalatha, Nuo Dong, Huping Wu, Zuguo Liu, Wei Li

**Affiliations:** 1 Eye Institute of Xiamen University, Xiamen, Fujian China; 2 Medical College of Xiamen University, Xiamen, Fujian China; 3 Fujian Provincial Key Laboratory of Ophthalmology and Visual Science, Xiamen, Fujian China; 4 The Affiliated Xiang’an Hospital of Xiamen University, Xiamen, Fujian China; 5 Xiamen University affiliated Chenggong Hospital, Xiamen, Fujian China; 6 Guilin Women and Children’s Hospital, Guilin, Guangxi China; 7 Zhengzhou Second Hospital, Zhengzhou, Henan China; 8 Xiamen University affiliated Xiamen Eye Center, Xiamen, Fujian China

## Abstract

Amniotic membrane (AM) has been widely used as a temporary or permanent graft in the treatment of various ocular surface diseases. In this study, we compared the epithelial wound healing and tissue remodeling after ocular surface reconstruction with intact amniotic membrane (iAM) or denuded amniotic membrane (dAM). Partial limbal and bulbar conjunctival removal was performed on New Zealand rabbits followed by transplantation of cryo-preserved human iAM or dAM. *In vivo* observation showed that the epithelial ingrowth was faster on dAM compared to iAM after AM transplantation. Histological observation showed prominent epithelial stratification and increased goblet cell number on dAM after 2 weeks of follow up. Collagen VII degraded in dAM within 2 weeks, while remained in iAM even after 3 weeks. The number of macrophages and α-SMA positive cells in the stroma of remodelized conjunctiva in the dAM transplantation group was considerably less. In conclusion, dAM facilitates epithelial repopulation and goblet cell differentiation, further reduces inflammation and scar formation during conjunctival and corneal limbal reconstruction.

## Introduction

Amniotic membrane (AM), the innermost layer of the placental membrane, consists of a simple epithelium, a thick basement membrane, and an avascular stroma^1,2^. AM was first utilized as a dressing material in ocular surgery by Rotth and Sorsby in 1946^3,4^, furthermore, Kim and Tseng in 1995 were the first to report the use of AM as graft^5^. Since then AM has been widely used for broad spectrum of ocular surface diseases. AM can be used both as a graft, sewn in place of corneal or conjunctival defect, or as a patch, sutured or glued against the ocular surface^6^, or sutureless amniotic membrane transplantation via ProKera^TM^, a contact lens like instrument^7^. As a temporary patch, AM has been applied in the treatment of acute chemical/thermal burns^8–12^, acute inflammatory and ulcerative stage of Stevens–Johnson syndrome (SJS)^13,14^. As a permanent graft, it has been used in the treatment of corneal persistent epithelial defects and ulcers, neurotrophic keratitis^15–18^, bullous and band keratopathy^19–24^, primary and recurrent pterygia, chemical injury^25^, symblepharon^26,27^, and SJS^28^. Various studies have proved that AM exerts potent anti-inflammatory^1,2,29–32^, anti-scaring^33,34^, anti-angiogenic^35^, and promoting epithelial wound healing functions in the recipient eyes (for reviews, see^2^).

AM was also broadly used as a carrier either as intact amniotic membrane (iAM) or denuded amniotic membrane (dAM) to expand corneal^36,37^, conjunctival^38,39^, and oral mucosal epithelial cells^40,41^ for ocular surface reconstruction purpose. There are numerous studies compared the characteristics exhibited by corneal epithelial cells on iAM and dAM. Limbal epithelial cell suspension cultured on dAM showed better stratification and cell junction formation than that on iAM^42^. Limbal epithelial cells on iAM expressed ΔNp63α exhibiting the ability to maintain high proliferative potential, whereas cells cultured on dAM were ΔNp63α negative^43^. The limbal epithelial explant cultured over iAM expressed the stem cell associated markers (ABCG2, p63) and showed reduced expression of the differentiation markers (Connexin 43 and cytokeratin K3/K12) when compared to limbal epithelial cells cultured over dAM, therefore corneal epithelial cells maintain stemness better when cultured on iAM^44^. PI3K/Akt/FKHRL1 extracellular signal transduction pathway was observed to be activated when human limbal explants were cultured on iAM, but not on dAM^45^. Limbal explant cultured on dAM was not capable of maintaining the structural integrity of cultured epithelial cells^46^. Therefore, it is generally accepted that corneal limbal explant cultured on dAM showed higher outgrowth rate than that on iAM^47,48^, whereas limbal epithelial cells expanded on iAM maintained the progenitor phenotype efficiently than dAM. However, the underlying molecular mechanism that cause this difference is elusive.

Both iAM and dAM was utilized as efficient carrier in epithelial tissue engineering, but dAM has never been used as graft in ocular surface transplantation. The major perspective of amniotic membrane transplantation (AMT) is to reconstruct the ocular surface by enhancing re-epithelialization^6^. AMT has been successful in restoring the ocular surface, reducing scarring and inflammation in case of acute ocular burns, SJS, etc.^49,50^. AMT as a standalone therapy was an effective treatment for partial limbal stem cell deficiency, whereby AM was considered as an *in vivo* carrier for limbal epithelial cell expansion^51^. Since iAM and dAM showed significant difference during *ex vivo* expansion of epithelial cells, we proposed the hypothesis that the process of re-epithelialization and tissue remodeling may vary when the ocular surface is reconstructed with iAM or dAM. In this study, we tested this hypothesis using rabbit partial ocular surface reconstruction model. We observed that dAM facilitated epithelial repopulation, goblet cell differentiation, reduced inflammation and scar formation during ocular surface reconstruction. The efficacy of dAM on tissue remodeling after transplantation is further discussed.

## Results

### Ocular Surface Wound Healing after AM Transplantation

**Figure 1:**
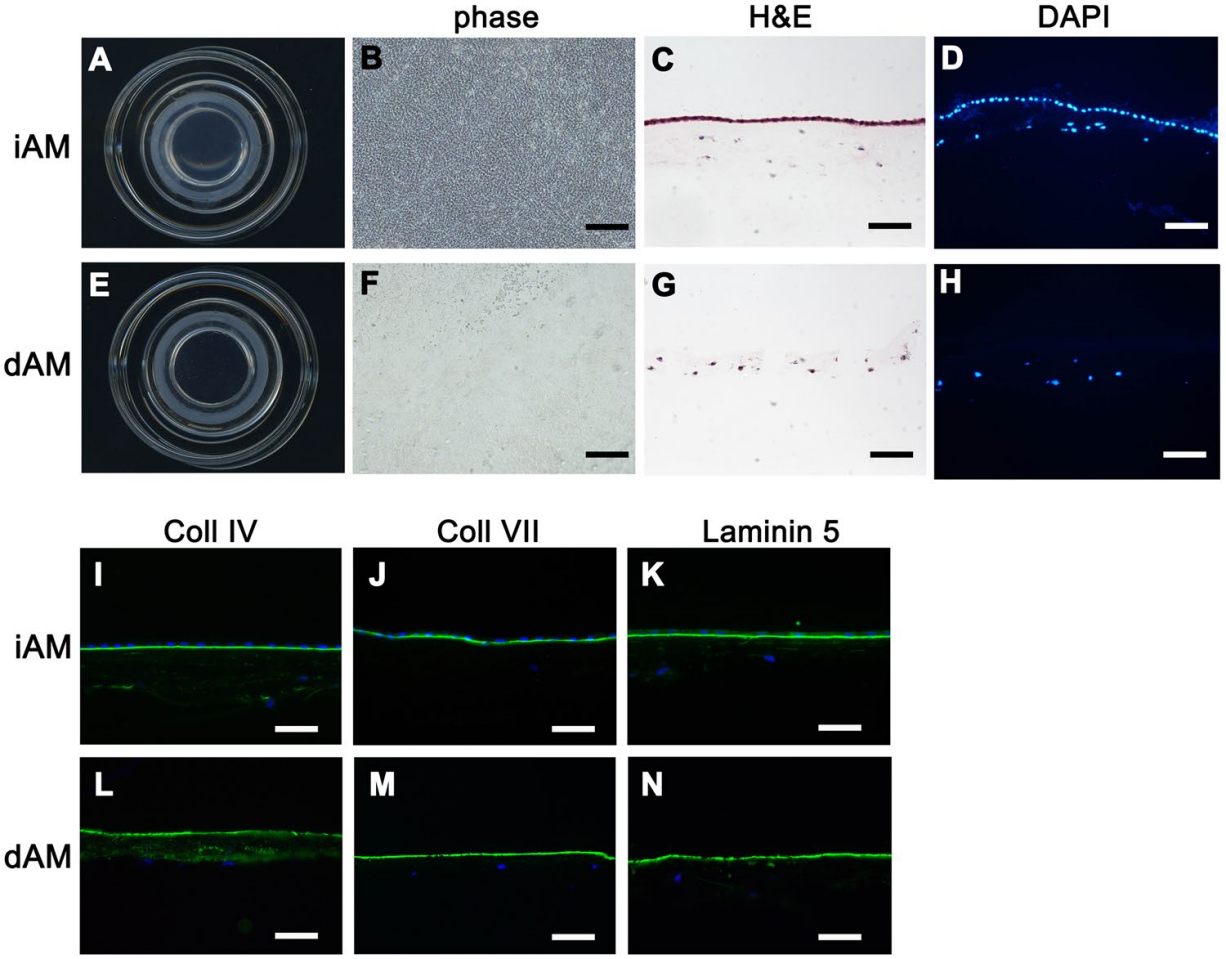
Macroscopic and microscopic evaluation of iAM and dAM. Cryopreserved iAM mounted on the culture insert showed sub-transparent appearance **(A)**. Phase contrast image of the iAM whole mount showed uniformly arranged and well maintained epithelial cells **(B)**. H&E **(C)** and DAPI staining **(D)** of iAM crosssection showed organized epithelial layer. Transparent dAM after 0.02% EDTA treatment and electric toothbrush scraping **(E)**. Phase contrast image of the dAM whole mount showed few epithelial cells **(F)**. H&E staining **(G)** and DAPI staining **(H)** of dAM crosssection confirmed the presence of an intact stroma without the epithelial layer. Immunostaining of collagen IV **(I**,**L)**, collagen VII **(J**,**M)** and Laminin 5 **(K,N)** showed all these basement membrane components were well preserved after amniotic membrane epithelial denudation. Bars represent 500 μm in (**B** and **F**), and represent 100 μm in (**C**,**D**,**G**,**H**, **I**–**N**).

Cryopreserved iAM showed subtransparent apparance after mounted on culture insert (Fig. 1A). Devitalized AM epithelial cells were well maintained under phase contrast whole-mount observation (Fig. 1B), which was confirmed by H&E staining (Fig. 1C) and DAPI staining (Fig. 1D). After 0.02% EDTA treatment and electric toothbrush scraping, the transparency of AM increased (Fig. 1E), which had few epithelial cells on the AM surface under phase contrast microscopic observation (Fig. 1F), H&E staining (Fig. 1G) and DAPI staining (Fig. 1H) further confirmed the absence of epithelial cell on the dAM surface and an intact stroma. Immunostaining of collagen IV (Fig. 1I,L), collagen VII (Fig. 1J,M) and Laminin 5 (Fig. 1K,N) showed all these basement membrane components were well preserved after amniotic membrane epithelial denudation.

**Figure 2:**
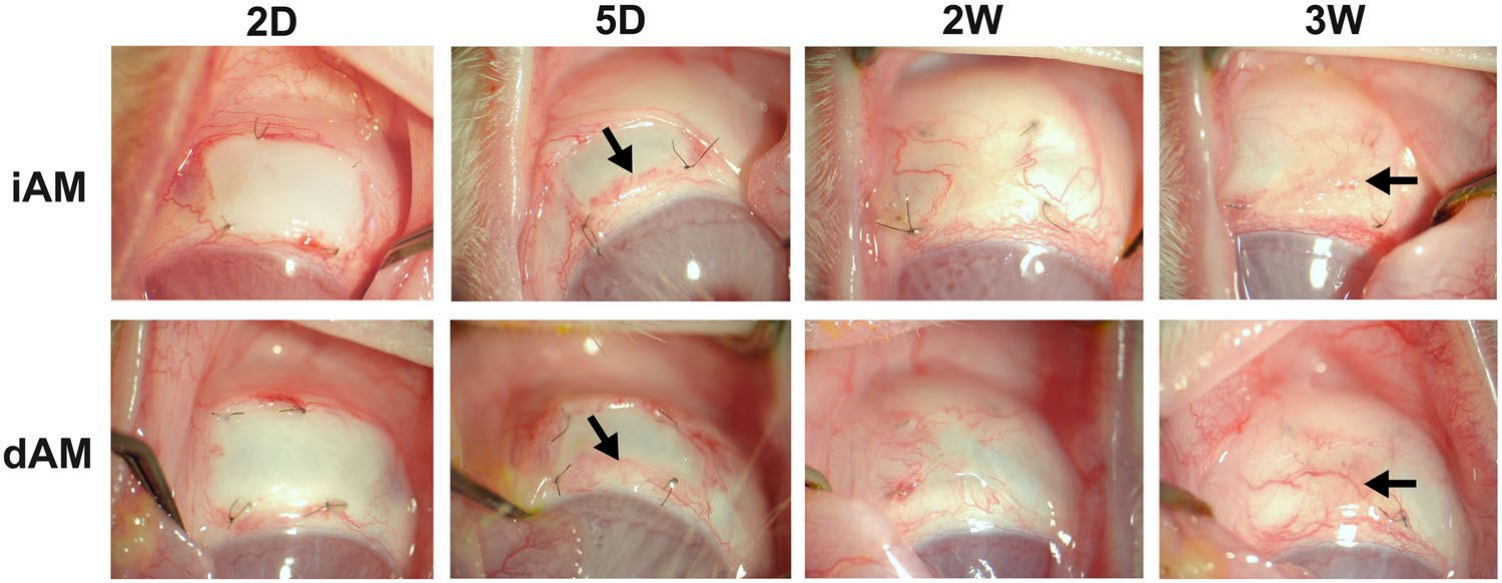
Evaluation of ocular surface changes in rabbit cornea transplanted with AM. Slit lamp microscopy images showed complete attachment of transplanted AM devoid of hemorrhage or injection under the AM in both iAM and dAM transplantation groups 2 days post surgery (2D). Five days (5D) followup showed the origin of neovascularization beneath the transplanted AM (arrow heads). Two weeks (2W) post surgery showed well vascularized AM similar to the surrounding host tissue. Three weeks (3W) followup showed thickened conjunctival tissue at the iAM transplantation loci, whereas dAM transplanted showed smooth and normal conjunctival tissue (arrow heads).

**Figure 3:**
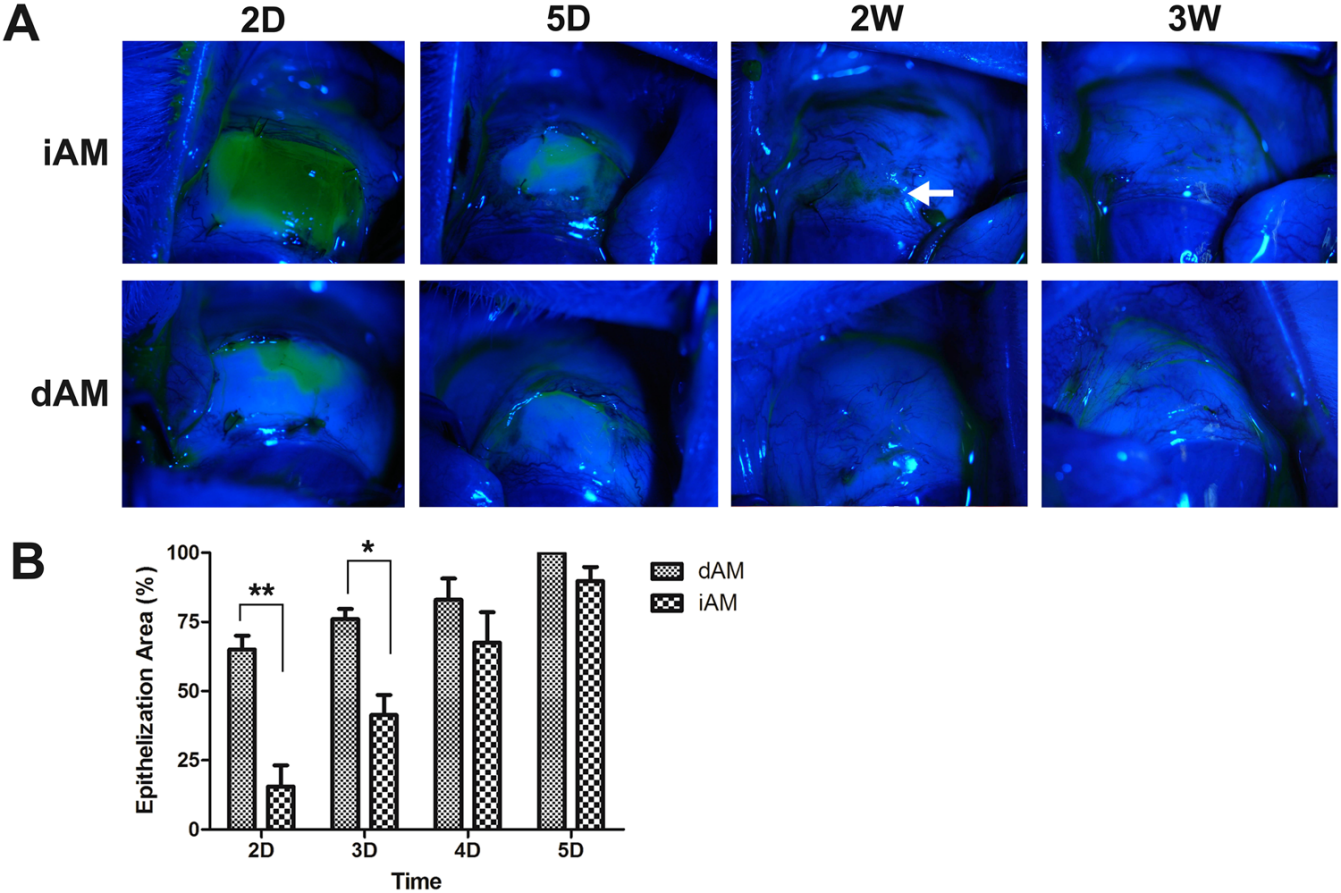
Evaluation of epithelialization using fluorescein corneal staining. Two days postsurgery, the iAM surface showed large area of fluorescein staining, whereas on dAM surface the fluorescein uptake was much smaller (2D). Five days after surgery, the central area of iAM surface showed positive staining, while the dAM surface was almost negative (5D). Persistent dots fluorescein staining was observed on iAM even after 2 weeks (2W, arrow**)**, while was completly negative on the dAM **(A)**. Statistical analysis showed the area of epithelialization on dAM *vs* iAM, there was significant difference in day 2 and day 3 **(B**, *p < 0.05, **p < 0.01**)**.

After the microscopic evaluation, the iAM and dAM were used for rabbit ocular surface reconstruction surgery. Two days after AM transplantation on rabbit limbal area, both iAM and dAM attached well, and there was no hemorrhage or injection under the AM (Fig. 2D). Five days after the surgery, neovascularization was observed underneath the AM that originated from the limbal area, the border between transplanted AM and the host conjunctiva was clear (Figs 1W and 2). Two weeks postsurgery, both iAM and dAM were vascularized, the density of blood vessel showed no dramatic difference between AM and surrounding ocular surface tissue, the border between transplanted AM and the host conjunctiva was not clear (Fig. 2W). Three weeks post surgery, mild increase in the thickness of the conjunctival tissue and rough surface at the iAM transplantation loci was observed. The new blood vessels underneath the conjunctival tissue were not clearly demonstrated under the slit-lamp microscope. In contrast, dAM transplanted area did not show conjunctival thickening, the conjunctival surface was smooth, and the blood vessels underneath the conjunctival were clearly showed (Figs 2 and 3W).

Fluorescein staining was performed to determine the epithelization of the AM surface. Two days post surgery, majority of the iAM surface was stained with fluorescence dye, however, fluorescence stained area was remarkably smaller on dAM surface. Spotted patterns of positive fluorescene staining persisted even after two weeks on iAM while it was negative on dAM (Fig. 3A). Statistic analysis showed that the area of epithelization on dAM was larger than that of iAM at day 2 and day 3 (Fig. 3B), indicating the epithelial wound healing was rapid on dAM than on iAM.

### Ocular Surface Tissue Remodeling after AM Transplantation

**Figure 4:**
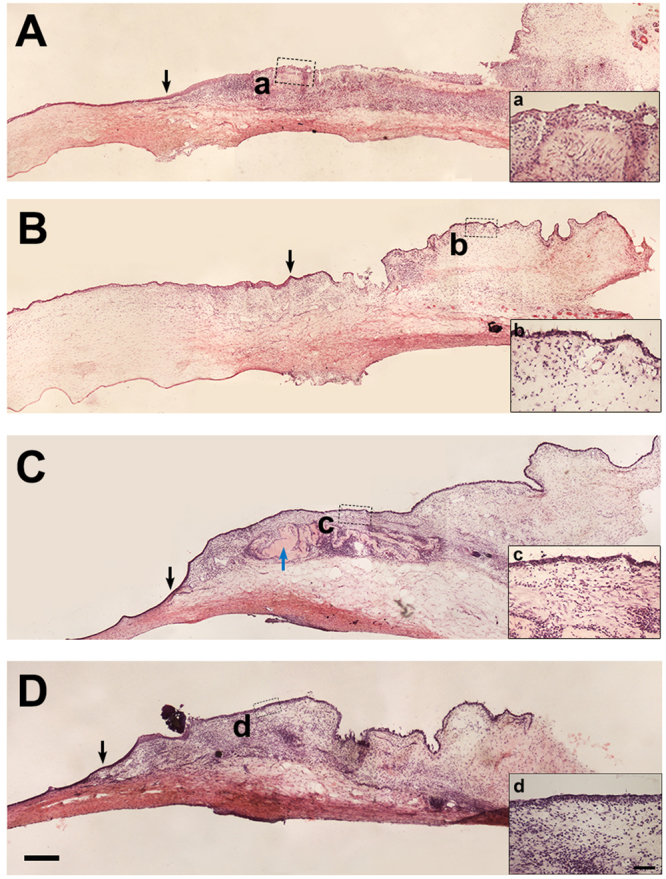
H&E staining on rabbit corneal and conjunctival tissue after AM transplantation. Visible stratified epithelium was observed 2 weeks post surgery with iAM **(A)** and dAM **(B)**. Uniform epithelial layer and intensified stromal cellularization was observed in iAM group 3 weeks post surgery. In addition, the stroma showed folded dense matrix like embedded structure **(C**, blue arrow head**)**. Three weeks post surgery sections of dAM transplanted group showed smooth and homogenous stroma **(D)**. Inserted figures show high magnification of the dotted portion (a,b,c,d). The black arrow heads show the corneal limbal junction. Bar represents 500 μm in (**A**–**D**), and represents 100 μm in a,b,c, and d.

**Figure 5:**
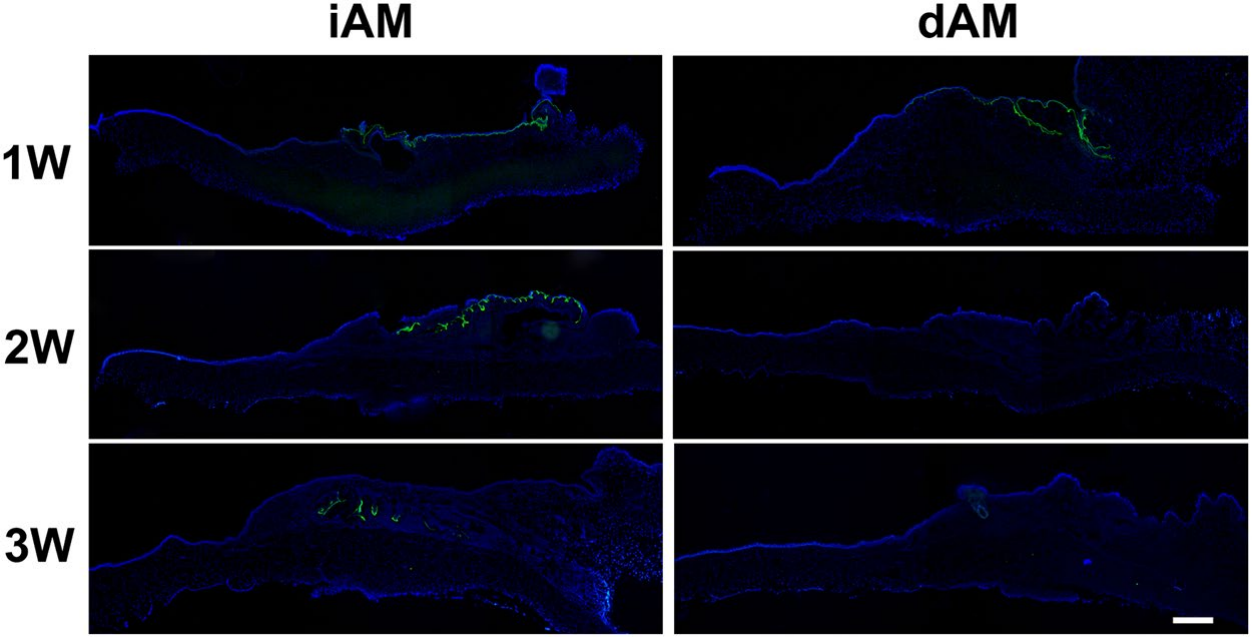
Anti-human type VII collagen immunostaining on corneal and conjunctival tissue after AM transplantation. Cross-sections of both iAM and dAM transplantation group showed strong type VII collagen staining after 1 week (1W). Cross-sections of iAM after 2 weeks (2W) shows type VII collagen specifically stained the folded matrix like structure under the iAM, whereas the sections of dAM group were negative. Three weeks (3W) sections showed that the positive staining sustained in the deep stroma of iAM group, indicating the presence of remenants of the iAM, however the dAM was completely negative for type VII collagen staining. Bar represents 500 μm.

H&E staining of the rabbit corneal and conjunctival tissue after AM transplantation was performed to demonstrate the tissue remodeling after AM transplantation. The results showed that 2 weeks post surgery, well stratified epithelium was observed on the surface of the transplanted area in iAM (Fig. 4A) as well as dAM (Fig. 4B) group. The stromal cellularization of the iAM group was obviously higher than that of dAM group. Three weeks after the surgery, the epithelium on the surface of the transplanted area were uniform and smooth in iAM (Fig. 4C) and dAM group (Fig. 4D). Interestingly, we found a folded matrix like tissue in the stroma of the surgery area in iAM group (Fig. 4C), while dAM group showed homogenous stroma (Fig. 4D). To further confirm the process of tissue remodeling post iAM or dAM transplantation, we performed immunostaining using antibody that specifically recognize human type VII collagen, which is one of the major components of AM basement membrane. As predicted, one week post surgery, strong positive staining was obsereved on the surface of the surgery area in both groups (Fig. 5). Two weeks after the surgery, type VII collagen staining showed folded matrix like structure under the surface epithelium in iAM group, however, there was no positive staining in dAM group. At 3 weeks, type VII collagen staining was positive in deep stroma of the surgery area in iAM group, fitting the position of matrix like tissue observed in H&E staining (Fig. 4C), while there was no staining in dAM group (Fig. 5). These results indicated that when ocular surface was reconstructed with iAM, the AM do not degraded completely and the remnants were gradually embedded into deep stroma of the regenerated host tissue. However, if the ocular surface was reconstructed with dAM, AM degraded within 2 weeks.

### Ocular Surface Epithelial Differentiation after AM Transplantation

**Figure 6:**
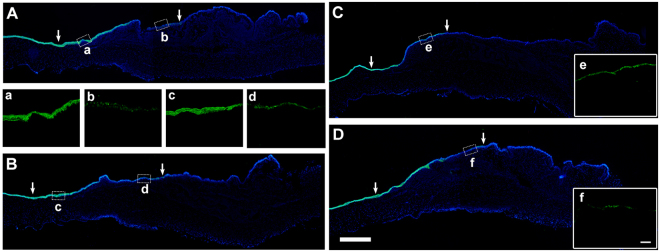
K12 immunostaining on ocular surface epithelium after AM transplantation. Two weeks after iAM **(A)** or dAM **(B)** transplantation, intense expression of K12 was observed in the border of the limbal area in iAM (a) as well as dAM group (c). The AM covered surgery area showed positive expression of K12 only in the superficial epithelial cells in both iAM group (b) and dAM group (d). Three weeks post surgery, K12 staining showed similar pattern in both iAM group **(C)** and dAM group **(D)**, with positive cells on the superficial layer of the regenerated epithelium on iAM (e) or dAM (f). Figure a–f showed high magnification of the dotted portions. The arrow heads showed the AM transplantation border of limbal side and conjunctival side. Bar represents 500 μm in **A**–**D**, and represents 100 μm in a–e and f.

**Figure 7:**
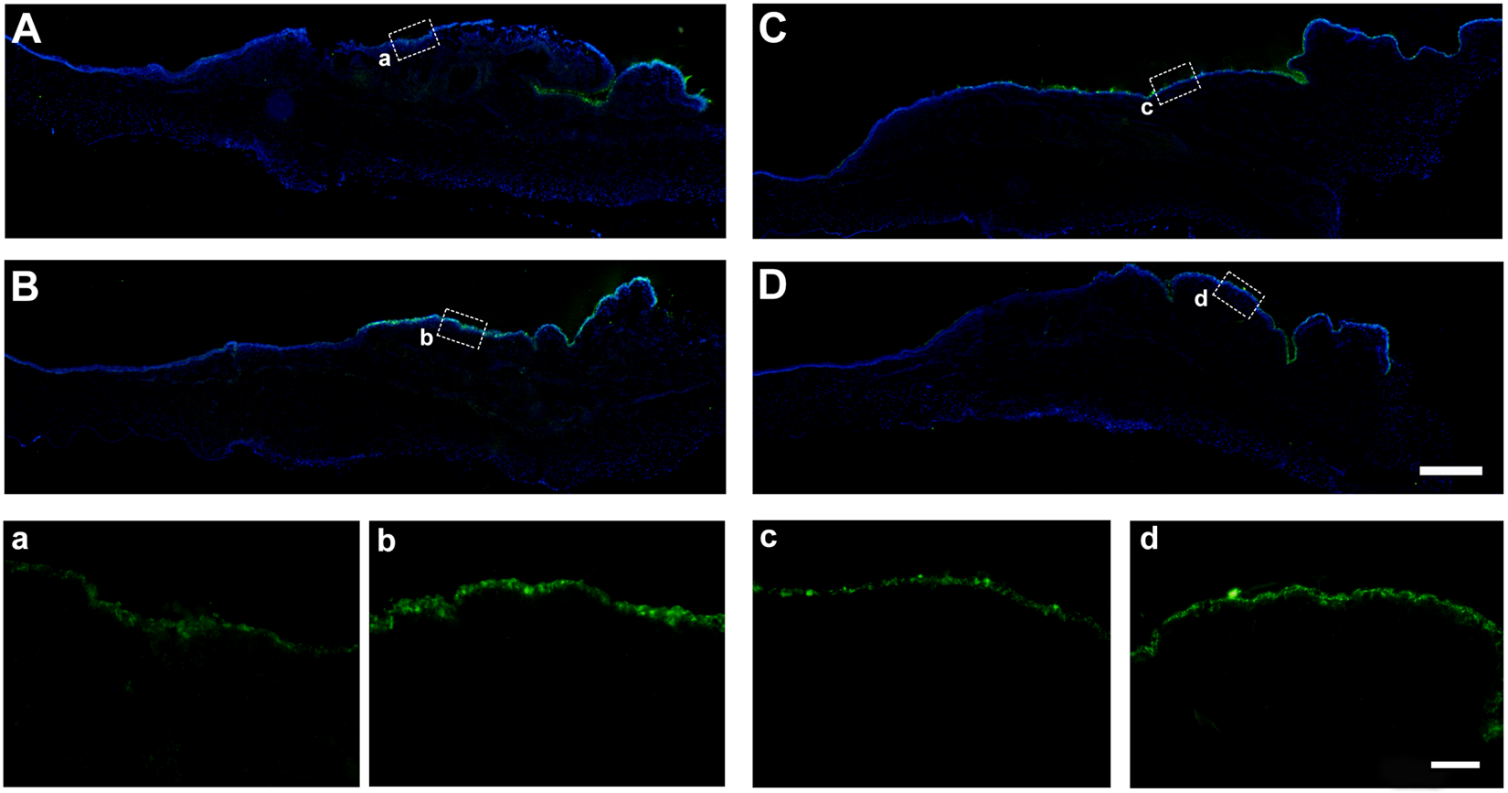
K19 immunostaining on ocular surface epithelium after AM transplantation. Two weeks after iAM **(A)** or dAM **(B)** transplantation, K19 positive staining was specific to the conjunctival surface of the transplantation area in both groups, the epithelium of the limbal area was K19 negative. High magnification images showed K19 expression was stronger on dAM group (b) than that on iAM group (a). Three weeks after iAM **(C**,c) or dAM **(D**,d) transplantation, K19 expression showed similar pattern without any significant difference. Bar represents 500 μm in (**A**–**D**), and represents 100 μm in a–d.

We then performed keratin K12 and K19 immunostaining, and PAS staining to investigate the epithelial differentiation status after iAM or dAM transplantation. K12 represents terminal differentiation of corneal epithelial phenotype, K19 represents conjunctival epithelial phenotype, while PAS staining reveals goblet cell phenotype. Two weeks after the surgery, the stratified epithelium in the AM border of limbal area showed strong expression of K12, in both iAM (Fig. 6a) and dAM group (Fig. 6c), while on the surface of AM that covered surgery area, the K12 positive staining was observed only on the superficial layer of epithelial cells in both iAM (Fig. 6b) and dAM group (Fig. 6d), indicating the incomplete differentiation of epithelial cells on the surgery area. Similar pattern was observed at 3 weeks post-surgery (Fig. 6C,D). K19 was expressed on the conjunctival side of the transplantation area in both groups, and showed no significant difference between 2 weeks (Fig. 7A,B) and 3 weeks (Fig. 7C,D).

**Figure 8:**
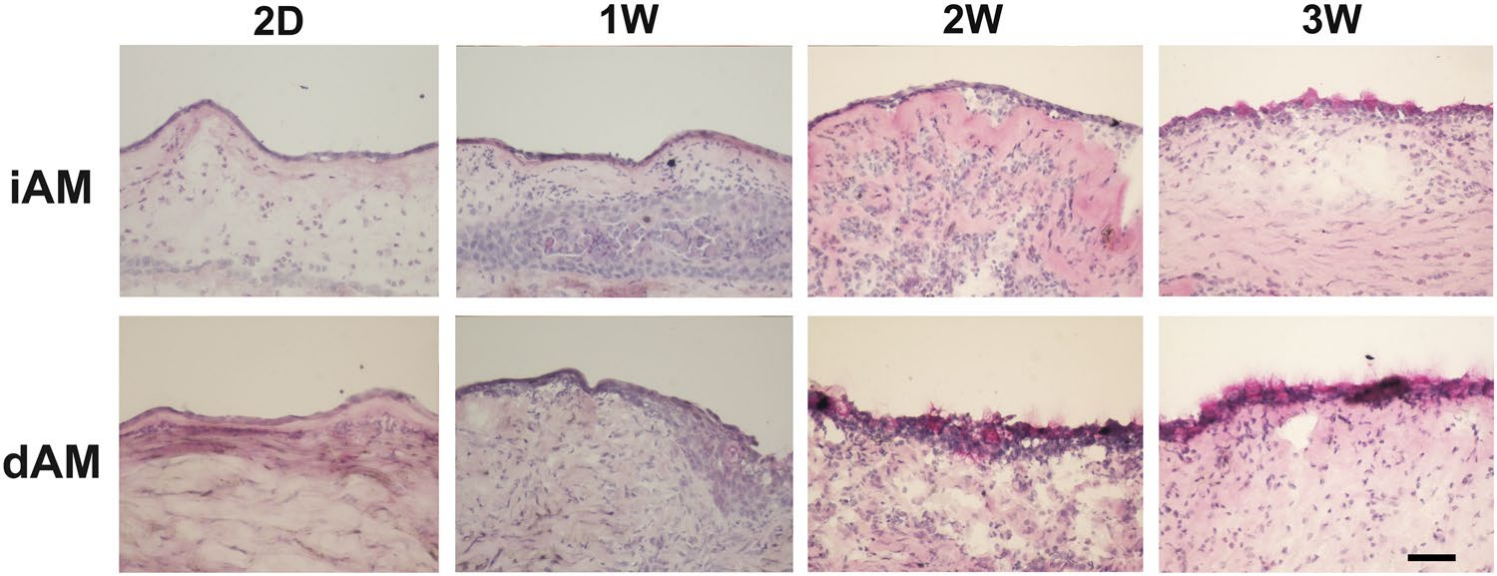
PAS staining on conjunctiva after AM transplantation. PAS staining was negative in the epithelium grown on iAM from 2 days to 2 weeks post surgery, PAS positive cells presented on the surface epithelium close to conjunctival side 3 weeks (3W) after iAM transplantation. However, PAS positive cells emerged on dAM 2 weeks after surgery and increased at 3 weeks. Bar represents 100 μm.

PAS staining showed the absence of goblet cell on day 2 on both iAM and dAM. PAS positive cells were observed on the conjunctival side of dAM one week post surgery and dramatically increased after 2 weeks. However, there was no PAS positive cells on iAM until 3 weeks. After 3 weeks, the number and pattern of goblet cells in dAM group were similar to that of the normal conjunctiva (Fig. 8).

### Stromal Inflammation and Scar Formation after AM Transplantation

**Figure 9:**
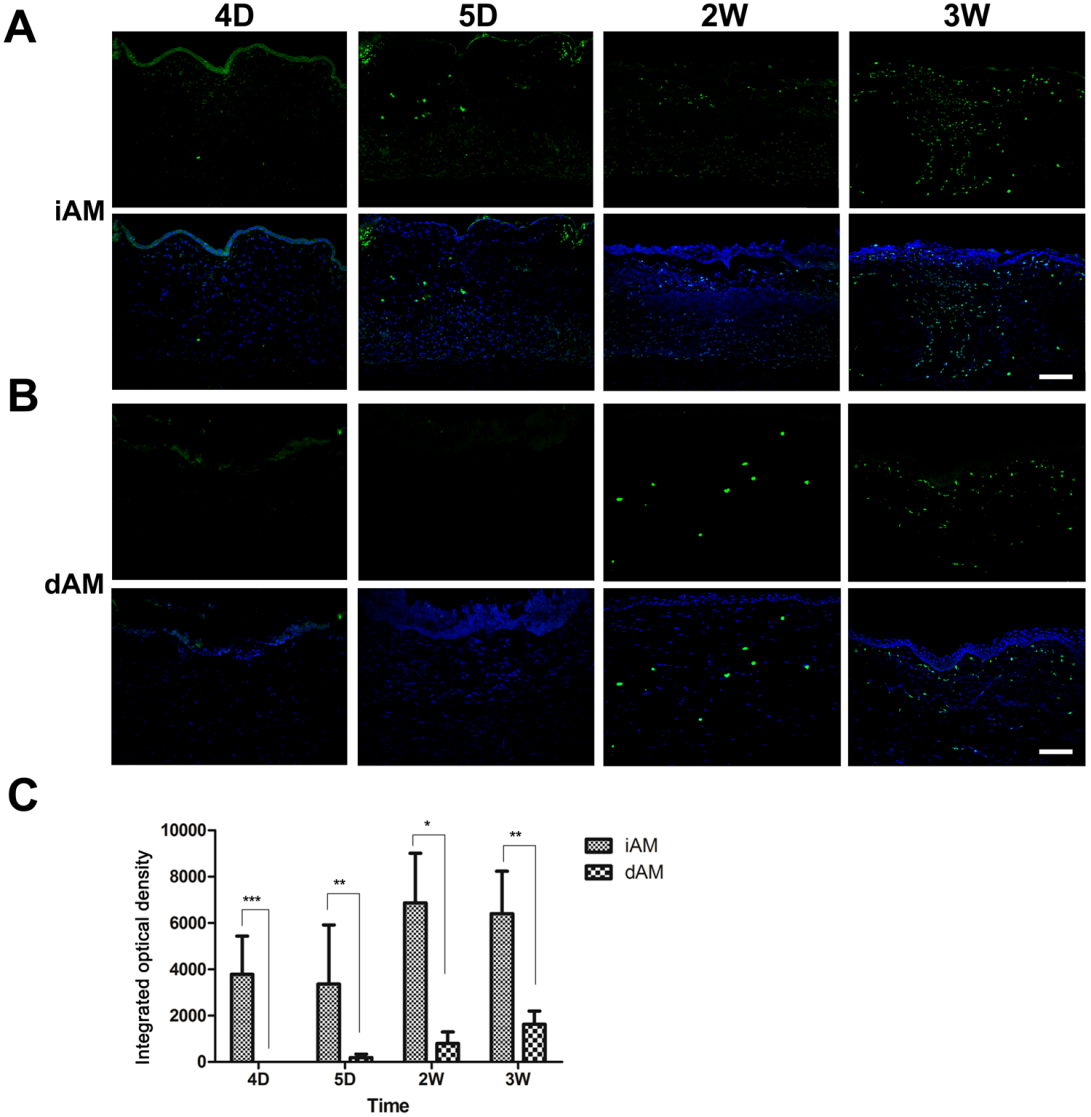
Macrophage infiltration in the AM loci after AM transplantation. Anti-macrophage antibody immunostaining (green fluorescence) showed infiltration of macrophages in the stroma of iAM transplantation loci **(A)** at 4 days (4D) that increased at 5 days (5D), and were found disseminated the entire stroma at 3 weeks (3 W). However, macrophage infiltration was absent in the dAM transplantation loci **(B)** 5 days post surgery, a few macrophages can be observed in the stroma 2 weeks post surgery (2W), and a gradual increase at 3 weeks (3W). DAPI staining (blue fluorescence) demonstrated cellularity of the tissues. Bar represents 20 μm. IOD analysis **(C)** showed significant difference in the rate of macrophage infiltration between the two groups post surgery at each time point **(B**, *p < 0.05, **p < 0.01, ***p < 0.001**)**.

Macrophage play the leading role during the intermediate and late stages of inflammation during wound healing. We investigated macrophage infiltration after the AM transplantation using macrophage specific antibody. Macrophages were observed in the stroma of iAM transplantation loci at 1 week and dramatically increased at 2 weeks, and dispersed throughout the stroma at 3 weeks (Fig. 9A). However, macrophage were absent in the dAM transplantation loci 1 week post surgery, a few macrophages emerged in the stroma 2 weeks after the surgery, and their numbers slightly increased at 3 weeks (Fig. 9A). IOD analysis confirmed the significant difference in the rate of macrophage infiltration between the two groups at all time points (Fig. 9B).

**Figure 10:**
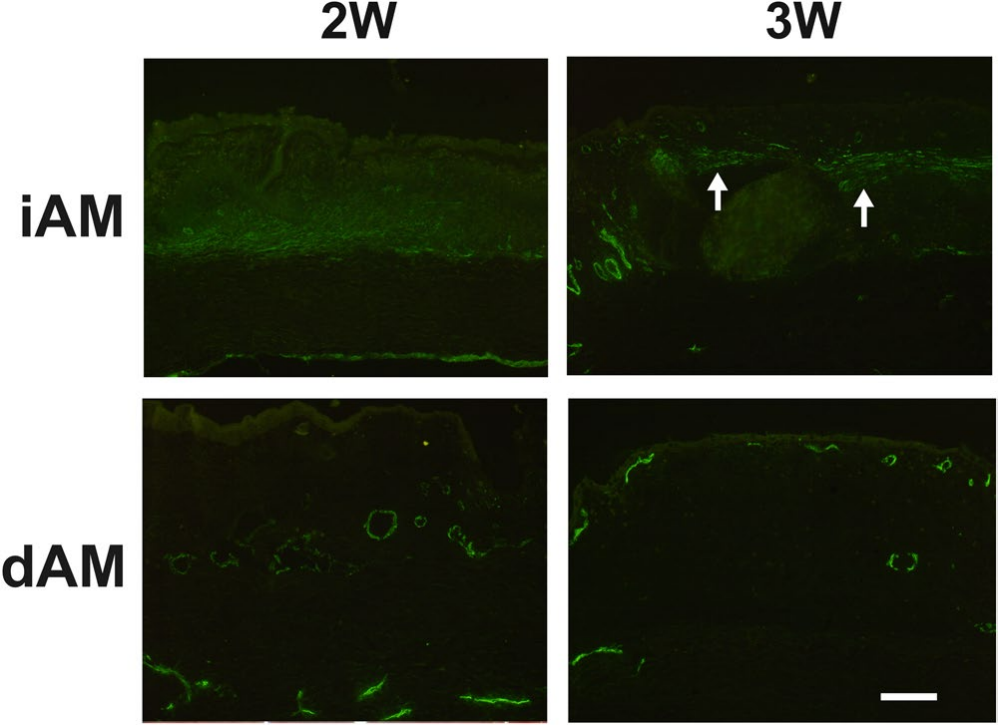
α-SMA immunostaining after AM transplantation. Two weeks (2W) post surgery, significant α-SMA positive cells were observed in the deep stroma of the iAM transplantation loci, and more intense staining was observed at 3 weeks (3W, arrow heads**)**. Negative staining pattern was observed in the dAM transplantation loci at 2 weeks and 3 weeks with the positive staining only presented in the blood vessels. Bar represents 200 μm.

To further investigate the wound healing after AM transplantation, we performed α-SMA immunostaining, marker of myofibroblast, that is present in the scared and fibrotic tissues. Two weeks after the surgery, α-SMA positive cells were presented in the deep stroma of the iAM transplantation loci, and the α-SMA expression became stronger and more condensed at 3 weeks. However, α-SMA positive cells were absent in the stroma of dAM transplantation loci at 2 weeks and 3 weeks other than blood vessel staining (Fig. 10). These data indicated that there was no scar formation and fibrosis after dAM transplantation.

## Discussion

This study compared the efficacy of iAM and dAM after *in vivo* transplantation on the basis of re-epithelialization and cell differentiation, inflammatory response, dissolution of basement membrane, fibrosis and scar formation. We found dramatic difference of tissue remodeling pattern after ocular surface reconstruction with iAM or dAM.

Firstly, the re-epithelialization of dAM after transplantation is rapid than that of iAM. Complete re-epithelialization was achieved within one week on transplanting dAM measuring 5 × 5 mm in rabbit. This was in consistence with *ex vivo* limbal explant culture, whereby outgrowth was hastened on dAM^47,52^. In dAM transplantation group, the basement membrane is directly exposed to the migrating epithelial cells. Previous study has shown that basement membrane of AM acts as a substrate supporting adhesion, growth, and differentiation of epithelial basal progenitor cells, facilitating migration of epithelial cells^53^. Studies have also demonstrated that in *ex vivo* limbal explant culture, the devitalized AM epithelial cells on iAM were disintegrated by matrix metalloproteinase produced by migrating epithelial cells and are removed during the process of epithelial cell expansion^47^. This procedure may hinder the migration rate of the epithelium. We presume that re-epithelialization of iAM after *in vivo* transplantation may follow similar machinery.

Basement membrane is important extracellular matrix which can facilitate epithelial cell attachment and migration. We found basement membrane components such as collagen IV, collagen VII and laminin 5 were well preserved on dAM after epithelium denudation. Our previous study proved that during *ex vivo* limbal explant culture on AM, the basement membrane components of AM dissolved within one week regardless of iAM and dAM^47^. In the current *in vivo* transplantation study, we found that type VII collagen, one of the major basement membrane components, was completely degraded at 2 weeks in dAM, whereas remained intact in iAM after 3 weeks. Therefore, *in vivo* dissolution of basement membrane components is delayed and there is difference between iAM and dAM. Our results also suggested that the basement membrane components of AM only act as temporary carrier or scaffold for epithelial migration, epithelial cells grown on AM eventually are capable of synthesizing and establishing their basement membrane.

Tissue remodeling procedure after tissue transplantation is critical to regain normal structure and physiological function^54^. Two weeks after AM transplantation, the graft loci became smooth in both groups. Based on the H&E staining results, we recognized that iAM was embedded into deep stroma of transplantation loci 3 weeks post surgery. This was also confirmed by type VII collagen staining. Such an integration of AM with the recipient tissue maybe beneficial if the purpose of the AM transplantation is for the conjunctival or fornix reconstruction. The embedded AM can act as tenon capsule or subconjunctival connective tissue. This concept has been applied in the treatment of conjunctivochalasis using AM to reinforce the strength of conjunctiva^55^. However, in the dAM transplantation group, we did not notice the remnant of dAM in the graft loci, and the cellularity of the graft loci stroma was quite homogeneous. Therefore, we presume that dAM was completely integrated into the host tissue, or it was completely degraded within 2 weeks. Contradictory conclusions are available in literature stating that transplanted AM survived and integrated into the host corneal tissue being modified or remodeled by the recipient cells^56^, while another study showed absence of AM remnants in recipient ocular surface months after AM transplantation in various diseases^24^.

The main purpose of AM transplantation for ocular surface reconstruction is to regenerate healthy epithelium. In rabbit models, we generated limbal stem cell deficiency and conjunctival defective rabbit model by partial limbal tissue and conjunctival tissue removal. We found that corneal epithelial differentiation was similar on iAM and dAM. It is generally accepted that epithelial cells expanded on iAM maintain more progenitor characteristic than that on dAM^42,47,48^. In other words, dAM promote differentiation of the epithelial cells. However, our *in vivo* data found that there was no dramatic difference of epithelial cell phenotype after transplantation of iAM or dAM. A major difference observed in our study is the presence of goblet cells on dAM 2 weeks after transplantation, indicating dAM may facilitate goblet cell differentiation.

Another intriguing finding of current study is that there was decreased macrophage infiltration in dAM group compared to iAM group, indicating dampened inflammatory response after dAM transplantation. As we know, re-epithelialization is the key procedure of epithelial wound healing. Inflammation tampers only after complete of epithelialization. Therefore, rapid re-epihtelialization in dAM group may be beneficial in reducing inflammation, that resulted in decreased macrophage infiltration. Another possibility of higher infiltration of macrophages in iAM may be due to the presence of devitalized AM epithelial cells. Disintegration of these cells may attract macrophages at the transplantation site to perform the function of dead cell scavenging. The embedded human AM tissue are capable of directly recruiting macrophages as they are rich source of human xeno-proteins. On considering the consequence of the xenoproteins, dAM may be the better choice in terms of reducing local inflammation.

Another supportive evidence is that after iAM transplantation, increased number of α-SMA positive cells accumulated in the stroma at the surgery loci. Since we can easily dissociate α-SMA positive blood vessels, the α-SMA positive cells should be the myofibroblasts, which are observed mainly in scar tissue. The macrophage can directly recruit myofibroblasts or through the secretion of inflammatory cytokines such as TGF-β that induces the transdifferentiation of fibroblast to myofibroblast^57^. Further investigation is required to prove whether the myofibroblasts originate from the blood circulation. We proposed that rapid re-epithelialization and degradation of AM tissue after wound healing may play critical role in the tissue remodeling after ocular surface reconstruction with dAM.

This study was performed in the ocular surface of healthy rabbits to investigate the ocular surface reconstruction procedure after AM transplantation. More intensive study should be conducted to compare the difference between iAM and dAM on ocular surface reconstruction under different conditions such as chemical burn or symblepharon. Although epithelial migration and healing is promoted on dAM, meantime, degradation is also hastened in dAM. Therefore, in ocular surface reconstruction of large defective area with obvious ocular surface inflammation, we do not recommend the use of dAM, as they degrade rapidly before complete epithelial healing that may lead to failure of the surgery. Future study is mandatory to address this query in animal model and clinical setting. Long term observation after surgery is also required to observe the outcome of the ocular surface reconstruction. Additionally, investigations to explain the mechanism of action behind the physiological properties of dAM continues to be of great interest according to our results.

In summary, our study for the first time conducted an *in vivo* study on the application of dAM on ocular surface reconstruction. Denudation of devitalized amniotic membrane epithelial cells facilitates epithelial repopulation and goblet cell differentiation, while further reduces inflammation and scar formation after transplantation. Our study may shed new light on the modification of amniotic membrane regarding its future application in clinic, dAM transplant could be a concomitant medical therapy in treating various ocular surface reconstructions.

## Materials and Methods

### Materials and Reagents

EDTA, Glycerin, 4′,6-diamidino-2-phenylindole I (DAPI), bovine serum albumin (BSA), mouse anti-type IV collagen antibody, mouse anti-human collagen VII antibody, and PAS kit were from Sigma (St. Louis, MO, US). Dulbecco’s modified Eagle medium (DMEM) without phenol red was from Invitrogen (Eugene, OR, US). Mouse anti-laminin 5 antibody, anti-cytokeratin 12 (K12) antibody, anti-cytokeratin 19 (K19) antibody, anti-vimentin antibody, anti-α smooth muscle actin (α-SMA) antibody, and mouse anti-macrophage monoclonal antibody were from Abcam (Cambridge, MA, US). FITC-conjugated anti–mouse IgG was from DAKO (Denmark).

### Preparation of Denuded Amniotic Membrane

With informed consent, in accordance with the tenets of the Declaration of Helsinki for research involving human subjects, and with the approval from the institutional review board of Medical College of Xiamen University, human AM was obtained at the time of cesarean section from a healthy human placenta, and prepared as per our previous report^58^. Immediately before use, AM was thawed, washed thrice with sterile PBS, and cut into pieces approximately 20 × 20 mm in size. For preparation of dAM, membranes were deprived of their devitalized amniotic epithelial cells by incubation with 0.02% EDTA at 37 °C for 1 hour, to loosen the cellular adhesion, followed by gentle scraping with a cell electric toothbrush, cut into 5 × 5 mm pieces and used for transplantation^47^.

### AM Transplantation and Post-operative Observation of Rabbits

Twenty four New Zealand white rabbits were obtained from Experimental Animal Center of Xiamen University, they were maintained and treated in accordance with the ARVO Statement for the Use of Animals in Ophthalmic and Vision Research and according to an experimental procedure approved by the Committee for Animal Research at Xiamen University. The animals were anesthesized with pentobarbital at the dosage of 50 mg/kg intraperitonealy partial limbal and conjunctival tissue removal (5 × 5 mm in size) was performed in the upper limbal region of the both eyes. The identical size of human dAM and iAM were transplanted to the corresponding limbal and conjunctival area of the left and right eye, respectively. The transplanted eyes were observed under slit-lamp microscope, and the fluorescein staining was performed everyday for one week post-operatively, and once a week thereafter until three weeks. Ocular surface and fluorescein staining images were taken under slit lamp microscope. At the time points of 2 days, 4 days, 5 days, 1 week, 2 weeks, and 3 weeks, four rabbits were sacrificed, the transplanted area including peripheral cornea, limbus, conjunctival, and underneath sclera were removed and embedded in optimal cutting temperature (OCT) compound for cryosectioning.

### Histology and Immunostaining

Cryostat sections (5 μm) of the rabbit tissues were fixed in acetone for 10 minutes at −20 °C. The sections used for immunostaining were rehydrated in PBS and then incubated in 0.2% Triton X-100 for 10 minutes. After three rinses with PBS for 5 minutes each and pre-incubation with 2% BSA to block nonspecific staining for 1 hour, the sections were incubated with primary antibodies (anti type VII collagen antibody at 1:400, others at 1:100) overnight at 4 °C. After three washes with PBS for 15 minutes, sections were incubated with an FITC-conjugated secondary antibody (chicken anti-mouse IgG at 1:100) for 45 minutes. After three additional PBS washes for 15 minutes, they were counterstained with DAPI (1:2000) for 1 minute, mounted in mounting medium and examined with a Nikon microscope (Nikon, Japan).

### Periodic Acid-Schiff (PAS) Staining

Cryostat sections (5 μm) of the rabbit tissues were fixed in 95% alcohol for 30 minutes at room temperature. After three rinses with double distilled water (DDW) for 5 minutes each, the sections were treated with 1% periodic acid for 8 minutes. After additional three rinses with DDW, the sections were stained with Schiff reagent for 5 minutes and suspended with DDW until purple appear in evidence. The sections were then counter-stained with hematoxylin for 2 minutes, cleared in xylene, covered with mounting medium and observed under light microscope.

### Statistical Analysis

Ocular surface fluorescein staining images representing epithelial healing process was analyzed using Image Pro Plus V6.0 (Media Cybernetics, Silver Spring, MD, USA) to calculate the relative surface area. Integrated optical density (IOD) of positive immuno-staining of anti-macrophage polyclonal antibody were also analyzed by Image Pro Plus V6.0 as previously reported^59^. Summary of the data were reported as mean ± SD. Group means were compared using the appropriate version of Student’s unpaired *t*-test, where *p* < 0.05 was considered statistically significant.
